# Electricity and natural gas tariffs at United States wastewater treatment plants

**DOI:** 10.1038/s41597-023-02886-6

**Published:** 2024-01-23

**Authors:** Fletcher T. Chapin, Jose Bolorinos, Meagan S. Mauter

**Affiliations:** Stanford University, 473 Via Ortega, Stanford, CA 94305 USA

**Keywords:** Environmental economics, Energy and society

## Abstract

Wastewater treatment plants (WWTPs) are large electricity and natural gas consumers with untapped potential to recover carbon-neutral biogas and provide energy services for the grid. Techno-economic analysis of emerging energy recovery and management technologies is critical to understanding their commercial viability, but quantifying their energy cost savings potential is stymied by a lack of well curated, nationally representative electricity and natural gas tariff data. We present a dataset of electricity tariffs for the 100 largest WWTPs in the Clean Watershed Needs Survey (CWNS) and natural gas tariffs for the 54 of 100 WWTPs with on-site cogeneration. We manually collected tariffs from each utility’s website and implemented data checks to ensure their validity. The dataset includes facility metadata, electricity tariffs, and natural gas tariffs (where cogeneration is present). Tariffs are current as of November 2021. We provide code for technical validation along with a sample simulation.

## Background & Summary

The water and wastewater sectors account for 3–5% of annual electricity consumed in the United States^1–3^. Electricity demand from wastewater treatment plants (WWTPs) often coincides with peak demand periods from other residential and commercial sectors^4^, resulting in higher marginal emissions and electricity prices^5^. Indeed, WWTPs typically consume 2.5 MWh per million gallons (or 0.65 MWh / ML) of wastewater treated^6–8^, and electricity typically accounts for 25–40% of the plant’s operational expenses^9^.

Faced with rising energy costs and decarbonization mandates, the wastewater community is exploring new resource recovery and energy management strategies. For example, electricity generated by combusting biomethane from sludge digestion can be stored in a battery to shift load to off-peak hours or participate in demand response markets^10^. While recent analyses suggest such approaches offer significant load shifting opportunities^11–15^, the magnitude of the bill savings and the net present value of any required infrastructure upgrades are a strong function of the prevailing electricity and natural gas tariffs.

The water and wastewater treatment community currently lacks a nationally representative dataset of electricity and natural gas tariffs, defined here as the overarching structure of charges (e.g., energy charges, demand charges) that determine the marginal price of energy delivered to a facility (i.e., inclusive of generation, transmission, and distribution charges). A collection of tariffs published by a single utility is called a tariff book. A recent working paper published by the International Monetary Fund (IMF) analyses country, regional, and global electricity consumption and cost data across all sectors, but the dataset lacks geographic granularity necessary for evaluating specific facilities^16^. High-resolution, wastewater sector-specific national^17^ and continental^18^ tariff datasets exist for Europe, but we are unaware of comparable datasets elsewhere. Instead, there are several localized (e.g., city or county-level^19^) electricity and natural gas tariff datasets that focus on specific markets (e.g., residential^20^) or provide energy charges in terms of average unit cost^21^. We seek to close this gap by providing a central repository of utility-published electricity and natural gas tariffs for the U.S. wastewater sector that explicitly accounts for underlying variability in charges as a function of time, location, and quantity of energy consumed.

The national dataset of electricity and natural gas tariffs we present here reflects the complexity of U.S. tariff structures. Tariffs typically have three components: (1) customer charges, or monthly minimum payments regardless of quantity of energy delivered, which vary by type of customer (e.g., residential, commercial); (2) energy charges billed by energy consumption in kWh or therms for electricity and natural gas, respectively; and (3) demand charges billed by monthly maximum electricity or natural gas usage in kW and therms/hr, respectively, and averaged over 5–15 minute increments. A therm is defined as 105.5 MJ, or the heat content of 100 cubic feet of methane at standard temperature and pressure. More complex tariffs may also have tiered energy and demand charges based on cumulative monthly demand, seasonal (i.e., monthly) and time-of-use (i.e., daily and/or hourly) variation in marginal energy and demand charges, and bundled or decoupled generation and delivery charges.

Within each component of the tariff, there is tremendous variability across both the underlying tariff structure and the charge per unit of energy delivered. As alluded to above, some utilities establish tariffs with flat rate energy and demand charges, where the price of energy is constant throughout the day. Other utilities establish tariffs with a time-of-use (TOU) component, where a peak hour surcharge is applied to each kWh (or therm) of energy delivered and kW (or therm/hr) of demand. TOU periods can vary from weekend to weekday in addition to hour-to-hour. Prices may also change seasonally (i.e., month-to-month) based on climate, with the highest prices depending on whether heating or air conditioning drive the maximum load on the grid. Finally, prices often vary based on total monthly consumption. For example, Con Edison charges almost $33/therm for the first 3 therms delivered and less than $1/therm thereafter^22^.

**Fig. 1:**
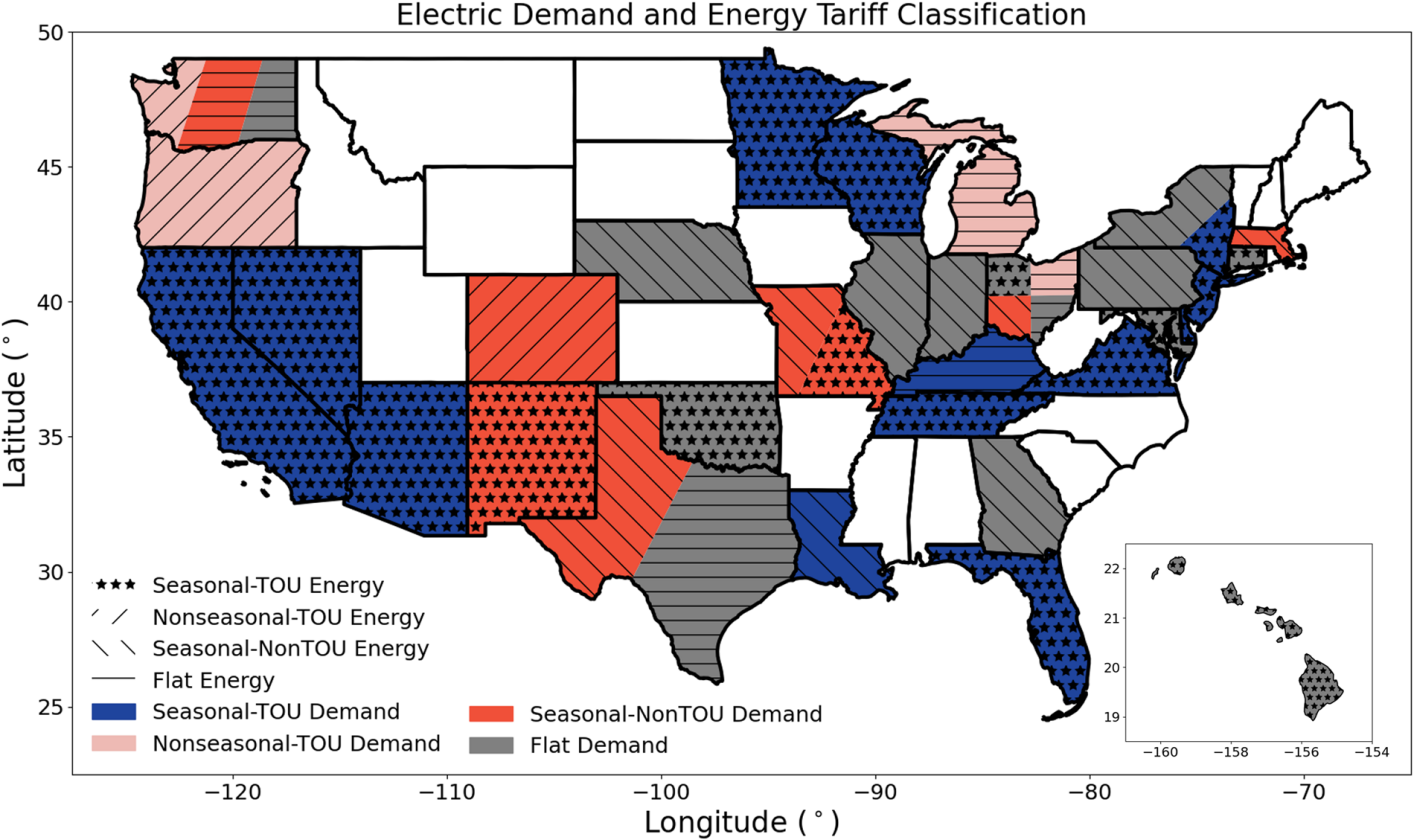
Map displaying the electric demand and energy tariff structures aggregated at a state level. Each facility’s tariff was categorized as seasonal time-of-use (Seasonal-TOU), nonseasonal time-of-use (Nonseasonal-TOU), seasonal non-time-of-use (Seasonal-NonTOU), or Flat. States with multiple facilities with different tariff structures are sliced to display the different tariff structures found throughout the state. The sizes and locations of each slice are not meaningful. Hawaii is not to scale. Alaska is not shown as none of the largest 100 facilities in the Clean Watershed Needs Survey^30^ are located in the state.

This dataset contains metadata and electricity tariffs from November 2021 for the 100 largest WWTPs in the United States (Fig. 1). Natural gas tariffs are included for the 54 of 100 WWTPs with on-site cogeneration. The tariffs, which were divided into customer, energy, and demand charges for both electricity and natural gas, were selected based on each facility’s estimated energy consumption from process-based models and previously published estimates of energy consumption as a function of treated volume. After collection, we validated the tariffs and metadata for completeness and consistency (see Technical Validation) before performing a sample analysis to illustrate how the dataset can be used (see Usage Notes). This dataset was originally gathered to enhance the external validity of Bolorinos *et al*.’s analysis of energy flexibility at a case study WWTP in California^23^.

The availability of a national electricity and natural gas tariff dataset has several critical applications. First, it will facilitate technoeconomic assessment of energy efficiency investments and load shifting activities at U.S. wastewater facilities. Second, it will facilitate analysis of the degree to which cost incentives for electricity reduction are aligned with a broader policy goal of water sector decarbonization^24,25^. Third, it will allow researchers to run wastewater process simulations with variable instead of average energy prices to achieve more realistic cost-benefit analyses^26^. Fourth, this dataset could complement broader analysis of the synergistic opportunities between diverse industrial energy loads and time-of-use electricity and natural gas tariffs to determine which sectors have the greatest energy flexibility or demand response potential^27^. Finally, the data could support siting analyses for cogenerators and heat recovery pumps^28,29^ critical to energy transition planning by providing GPS coordinates of existing cogenerators that are prime candidates for heat pump installation.

## Methods

### Scope of Analysis

**Fig. 2:**
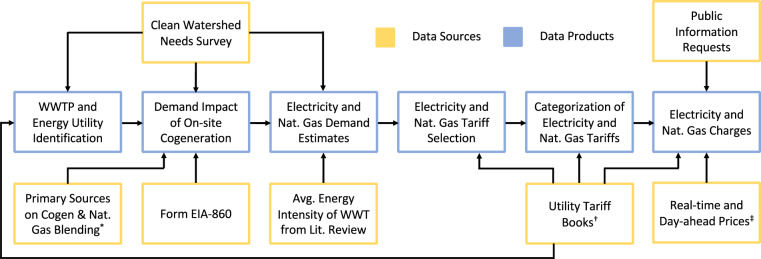
Flow chart illustrating data collection and production methods. ^*^Primary sources include air quality permits, engineering drawings, case studies, facility inspections, and press releases. ^†^See Tables 2–4 for utility tariff book references. Public information requests were necessary in Houston and Dallas where electricity tariffs were not published online. ^‡^See Table 5 for full list of sources for real-time and day-ahead prices. Facilities were identified by the Clean Watershed Needs Survey (CWNS)^30^. Electric and natural gas utilities were identified according to service area maps or ZIP codes from utility tariff books The presence of cogeneration was determined from a combination of the CWNS^30^, Form EIA-860^21^, and primary evidence^22^, such as air quality permits. The presence of cogeneration, flow rates from the CWNS, and wastewater treatment (WWT) energy intensity (2.5 MWh / million gallons)^6–8^ were then used to estimate the electric grid and natural gas demand of each facility. Finally, the appropriate electricity and natural gas (if applicable) utilities were selected according to each facility’s CWNS address, and tariffs were assigned by estimated demand. Natural gas tariffs were selected only for facilities with on-site cogeneration. A combination of utility tariff books, historical real-time and day-ahead prices, and public information requests was used to determine the charges within each tariff.

This dataset contains metadata and electricity tariffs from November 2021 for the 100 largest WWTPs in the United States (Fig. 2). Natural gas tariffs are included for the 54 of 100 WWTPs with on-site cogeneration. For each WWTP, we determined the presence of on-site biogas cogeneration, estimated facility-level electricity demand, and estimated natural gas demand if cogeneration was present. These demand estimates were then used to select the appropriate tariff for each WWTP, as many tariff books are stratified based on monthly consumption. Next, we classified the relevant tariffs by their monthly and daily temporal variation and extent to which generation and distribution charges are bundled. Finally, we collected the electricity and natural gas charges at each of the 100 largest WWTPs.

### Wastewater Treatment Plant and Energy Utility Identification

We used the Clean Watersheds Needs Survey (CWNS)^30^ to determine the 100 largest WWTPs in the United States by flow rate. The electric and gas utilities serving these WWTPs were determined by facility address and utility service area documentation contained in the utility tariff book.

### Demand Impact of On-site Cogeneration

Facilities with on-site cogeneration can offset substantial portions of their electricity and natural gas demand^31^. Thus, it is necessary to accurately establish the presence of biogas production and combustion on site before estimating the grid electricity and natural gas demand of a facility. We collected and compared this data using three distinct sources. The CWNS dataset identified 15 facilities with cogeneration^30^. We cross-referenced with data from the Energy Information Administration (EIA) Form 860 – Schedule 3 ‘Generator Data’ for generators fueled by either ‘Landfill Gas’ or ‘Other Waste Biomass’ in the states and counties corresponding to our 100 WWTPs. We manually matched the “Utility Name” and “Plant Name” fields to identify WWTPs in our sample with cogeneration^21^. In total, we found 24 facilities with cogeneration in the EIA dataset that were not indicated as having cogeneration in the CWNS. This included 2 EIA entries that were mislabeled as ‘Natural Gas Fired Combined Cycle’ or ‘Natural Gas Fired Combustion Turbine’ but corresponded to a WWTP with biogas cogeneration.

Unfortunately, even the processed EIA data did not capture all WWTPs with cogenerators. For example, Form EIA-860 only includes data on engines with at least 1 MW of capacity^21^ and some facilities operate smaller engines^22^. Our filtering methodology may also miss facilities with cogenerator operations contracted out to a third party^32^. We thus conducted Google searches for primary sources, such as air quality permits from local^33–35^ and state^36–39^ monitoring authorities, documentation on municipal websites^40–44^, state inspections^45^, technical reports^46^, EPA case studies^47,48^, and press releases^49–52^, and used those sources to flag additional facilities with cogeneration. We identified 15 additional facilities with on-site cogeneration that were not documented in the CWNS or the Form EIA-860 and documented those sources in reference_list.csv, for a total of 54 of 100 facilities with cogeneration^22^.

The performance of cogenerators at individual facilities is highly variable and proprietary. As a result, we use average literature values to estimate electricity and natural gas demand reduction. We assume that the presence of a cogenerator reduces 50% of a WWTP’s electric power demand. We also assume that the cogenerator system meets 100% of the WWTP’s heat demand^31,53–55^, such that all facility natural gas consumption occurs in the cogenerator.

We assume that facilities operate their cogenerator to be compliant with local air emissions permits that establish an upper limit on the fraction of natural gas combusted (f_ng_). We reviewed a nationally representative subset of seven cogenerator air quality permits (out of 54 total facilities with cogeneration in our dataset) and identified f_ng_ values ranging from 4.4%^39^ and 40%^33^, with a median value of 10%^34–38^. Thus, we assumed that f_ng_ = 0.1 when estimating existing and design natural gas demand (“Est. Existing Natural Gas Demand”; “Est. Design Natural Gas Demand”) and perform sensitivity analysis on this assumption to ensure that natural gas tariff selection remains unchanged over this range.

### Electricity and Natural Gas Demand Estimates

Electricity and natural gas tariffs are often partially determined by the peak demand (or load). To select the correct tariff, we first estimate gross WWTP electricity demand, then estimate the quantity of electricity produced by cogeneration along with the natural gas imported for that cogeneration, and finally subtract the gross electricity demand from on-site electricity cogeneration to determine the WWTP’s net electricity demand. We approximate the electricity demand for each facility using Eq. (1),

1 $${{\rm{D}}}_{{\rm{g}}{\rm{r}}{\rm{o}}{\rm{s}}{\rm{s}}}={{\rm{E}}}_{{\rm{i}}}\ast {\rm{Q}}\text{}/(24\,{\rm{h}}{\rm{r}}/{\rm{d}})$$

where D_gross_ is the estimated gross electricity demand by the facility in MW, E_i_ is the average electric energy intensity of United States wastewater treatment, 2.5 MWh / MG^6–8^, and Q is the annual peak flow rate in million gallons per day (MGD). The value selected for E_i_ is within the range of several recent estimates of E_i_ in the peer reviewed literature^6–8^, but this value is likely to vary moderately on a facility basis depending on the level of treatment and the efficiency of plant operations. As explained further in “Validation with Existing Data”, we do not expect these variations to significantly alter the tariff selection of these facilities.

There is also uncertainty and variability associated with the flow rate data across the 100 facilities in this study. The CWNS reports ‘existing total flow’, representing an average annual flow rate, and ‘design flow’, representing the theoretical maximum flow rate for which the plant was designed. In reality, the design flow is likely to fall below the actual maximum flow at plants treating significant wet weather flows. We resolve this issue by including electricity and natural gas demand estimates for both existing total flow and design flow in metadata.csv^22^. Examining a small sample of three facilities in California for which flow data was accessible, we found that design flow was closer to the annual peak flow than existing total flow. Therefore, we used design flow to compile our data set.

As a sensitivity analysis, we then applied the same methods using existing total flow. Despite the up to 54% difference between design flow and existing total flow, there were only four cases where using existing total flow would have resulted in a different tariff. In other words, tariff selection is robust to changes in assumed treatment flow rate.

We then use Eqs. (2–4) to estimate the net electric power and natural gas demand of each facility. We describe any simplifying assumptions unique to select facilities in WWTP_Billing_Assumptions.xlsx^22^.

2 $${\mathbb{1}}({\rm{C}}{\rm{o}}{\rm{g}}{\rm{e}}{\rm{n}})=\{\begin{array}{cc}1 & {if\; a\; facility\; has\; cogeneration}\\ 0 & {otherwise}\end{array}$$

3 $${{\rm{D}}}_{{\rm{el}}}={{\rm{D}}}_{{\rm{gross}}}{\rm{/}}(1+{\mathbb{1}}({\rm{Cogen}}))$$

4 $${{\rm{D}}}_{{\rm{n}}{\rm{g}}}={\mathbb{1}}({\rm{C}}{\rm{o}}{\rm{g}}{\rm{e}}{\rm{n}})\ast {{\rm{f}}}_{{\rm{n}}{\rm{g}}}\ast {{\rm{D}}}_{{\rm{e}}{\rm{l}}}\ast 34.12[({\rm{t}}{\rm{h}}{\rm{e}}{\rm{r}}{\rm{m}}{\rm{s}}/{\rm{h}}{\rm{r}})/{\rm{M}}{\rm{W}}]$$

In Eq. (3), D_el_, the estimated demand on the electric grid in MW (i.e., net electricity demand), is either set to D_gross_ or D_gross_/2 depending on Eq. (2), an indicator function for the presence of cogeneration. Past work suggests that facilities with cogeneration produce, on average, half of the consumed electricity on-site^55^. Finally, we use Eq. (4) to estimate the facilities gross demand for natural gas, D_ng_, in therms/hr, where f_ng_ = 0.1 as described above, and 34.12 is the conversion factor between therms/hr and MW (based on 105.5 MJ per therm).

In summary, for each facility we calculated gross electricity demand using Eq. (1), net electricity demand using Eq. (3), and natural gas demand for cogeneration using Eq. (4). The net electricity demand and natural gas demand were then used to select the appropriate tariffs. Since the goal of the original study was to investigate electric load shifting in WWTPs, natural gas tariffs were collected only for facilities with cogeneration^23^. In other words, natural gas usage when performing demand response with WWTPs will not change in the absence of cogeneration^56^, so natural gas tariffs were ignored in those cases.

### Electricity and Natural Gas Tariff Selection

We identify the relevant electricity and natural gas tariff from the appropriate utility tariff book by addressing three questions: (1) what type of entity is consuming energy? (2) what is the monthly peak demand? (3) what is the total monthly energy delivered?

Customer charges are typically based on whether the consumer is classified as residential, commercial, or industrial. Wastewater facilities are industrial customers. We selected a ‘General Service’ tariff in instances where industrial classifications did not exist.

In addition to customer type, energy and demand charges are also a function of monthly peak demand and total monthly energy delivered. Using the previously discussed methodology, we obtained two estimates for each facility’s demand, based on CWNS existing total and design flows. This demand was multiplied by the number of hours in a month to estimate the total monthly electricity or natural gas delivered in kWh or therms (respectively). Within the appropriate customer section (‘Industrial’ or ‘General Service’), we selected the tariff corresponding to the facility’s estimated electricity and natural gas energy and/or demand estimates. For example, we selected Industrial/General Service B-20 for Pacific Gas & Electric from a long list including Residential TOU, Commercial/General Service A-1, Commercial/General Service B-10, Streetlight Rates, etc.

Given the uncertainty in f_ng_ and the resulting impact on natural gas demand, we performed sensitivity analysis to ensure that the tariff selection remained unchanged with more extreme blend limits. In this dataset, natural gas tariff cutoffs were sufficiently broad such that varying the blend between 4.4% and 40% did not change the tariff selected for any facility. The tariff classification changed for the first facility (CWNS No. 25000128001) when the f_ng_ was increased to 47%.

### Categorization of Electricity and Natural Gas Tariffs

The applicable tariffs varied widely across the 100 WWTP in our dataset. To summarize their attributes, we descriptively categorized tariffs on two dimensions: (1) the hourly, daily, and monthly variability of energy and demand charges (“temporality”), and (2) whether electric power generation and delivery charges are bundled (“bundling”).

#### Temporality

We categorize monthly electricity energy and demand charges into Flat, Seasonal-TOU, Nonseasonal-TOU, or Seasonal-NonTOU charges:

1. Flat Charges: Charges are constant throughout the year.
2. Seasonal-TOU Charges: Charges vary monthly (Seasonal) and daily and/or hourly (TOU).
3. Nonseasonal-TOU Charges: Charges are consistent from month to month, but vary daily and/or hourly.
4. Seasonal-NonTOU Charges: Charges vary monthly, but are constant from day to day and hour to hour.

**Fig. 3:**
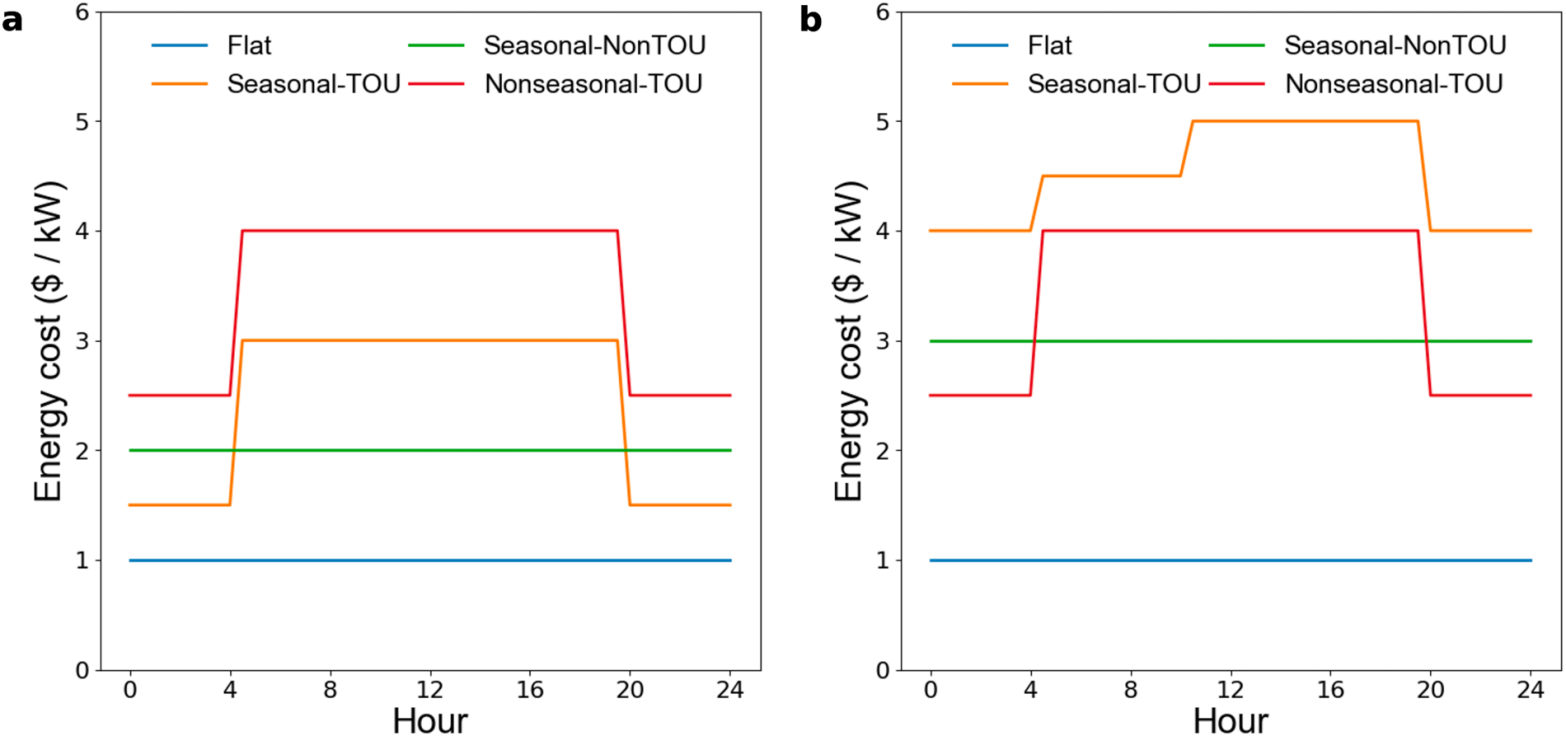
Representative flat, time-of-use (TOU), and seasonal price profiles. **(a)** Winter prices and **(b)** summer prices representing Flat, Seasonal-TOU, Nonseasonal-TOU, and Seasonal-NonTOU tariffs. Cost would be calculated in $/kW, $/(therm/hr), $/kWh, or $/therm for electricity demand charges, natural gas demand charges, electricity energy charges, and natural gas energy charges, respectively. A therm is defined as 105.5 MJ, or approximately the heat content of 100 cubic feet of natural gas.

We visualize this categorization using dummy charge data for winter and summer months in Fig. 3a,b, respectively. The seasonal patterns depicted in Fig. 3 represent the majority of the municipalities surveyed, but exceptions exist in select locations. For example, in Delaware the electricity energy charges were higher in winter than summer months. The partial peak seen in the Seasonal-TOU summer price (Fig. 3b) is another nuance of some tariffs, such as those of Pacific Gas & Electric, where there are multiple TOU periods with different charges. While not explicitly visualized, there may also be seasonal changes in the timing of peak periods. For example, Chattanooga, has winter morning peak and an afternoon/evening summer peak^22^. The temporality categories for electricity tariffs in our dataset are plotted in Fig. 1.

#### Bundling

In the United States, generation, transmission, and distribution charges are commonly bundled into a single electricity energy charge. However, in some cases facilities pay separate generation (or supply) and delivery (or transmission and distribution) charges to a single provider or to generation and delivery providers separately. For example, in much of the Texas interconnection, delivery and generation service providers are distinct.

We developed a second categorization scheme for electricity tariffs based on the extent to which generation and delivery charges are bundled:

1. Class 1: Fully bundled generation (supply) and delivery (transmission and distribution) charges.
2. Class 2: Bundled generation and delivery charges with fuel cost adjustment based on the market price of fossil fuels.
3. Class 3: Generation and delivery service from the same provider with pre-defined charges.
4. Class 4: Generation and delivery service from the same provider with pre-defined delivery charges and variable generation charges that reflect real-time or day-ahead electricity prices set by the live market.
5. Class 5: Generation and delivery service providers are legally required to be separate, and delivery charges are directly negotiated between customers and utilities. As a result, delivery charges are not published in tariff books, but can be obtained through public information requests.

We categorize each of the 56 electric utilities in our dataset in Table 1. For Class 2, ex-post analysis of our dataset revealed that the fuel cost adjustment would have accounted for between 0.5%^57^ and 60%^58–64^ of the total electricity bill of facilities in 2021.

**Table 1:** The classification of the 56 electricity utilities in our dataset based on the extent to which charges were bundled in the electricity tariff.

Electric Utility	Class	Number of Facilities	Electric Utility (cont.)	Class (cont.)	Number of Facilities (cont.)
TECO	2	1	AEP Ohio	3	2
Austin Energy	1	1	DTE Energy	1	2
Omaha Public Power District	2	1	Evergy	2	1
PSEG Long Island	3	3	Toledo Edison	3	1
Eversource (CT)	3	1	Rochester Gas & Electric	4	1
Pacific Gas and Electric	1	6	Georgia Power	2	1
PNM	2	1	Nashville Electric Service	2	1
CPS Energy	2	1	Entergy New Orleans	2	1
Public Service Electric And Gas	4	4	PECO	4	3
Southern California Edison	3	6	CenterPoint Energy	5	1
Con Edison	4	12	Louisville Gas and Electric	2	1
Dominion Energy	1	2	Seattle City Light	1	1
Oklahoma Gas & Electric	2	1	Ameren	2	2
Tacoma Power	1	1	AES Indiana	2	1
NV Energy	1	2	Cleveland Public Power	1	1
Portland General Electric	1	1	Salt River Project	1	1
EPB of Chattanooga	3	1	Duke Energy	3	1
Baltimore Gas and Electric	3	2	Xcel Energy (CO)	2	1
Penelec	4	1	Duquesne Light Company	4	1
Puget Sound Energy	1	1	SMUD	1	1
Memphis Light, Gas, and Water	2	2	The Illuminating Company	3	1
National Grid	4	2	Jersey Central Power and Light	4	1
AES Ohio	3	1	San Diego Gas & Electric	3	1
Florida Power and Light	1	4	Xcel Energy (MN)	2	1
Delmarva	1	1	We Energies	2	1
Hawaiian Electric	2	1	Comed	4	3
Oncor	5	4	Eversource (MA)	3	1
Ohio Edison	3	1	Pepco	3	1

The classification system was as follows Class 1 = fully bundled generation (supply) and delivery (transmission and distribution) charges; Class 2 = bundled generation and delivery charges with fuel cost adjustment based on the market price of fossil fuels; Class 3 = generation and delivery service from the same provider with pre-defined charges; Class 4 = generation and delivery service from the same provider with pre-defined delivery charges and variable generation charges that reflect real-time or day-ahead electricity prices set by the live market; and Class 5 = generation and delivery service providers are legally required to be separate, and delivery charges are directly negotiated between customers and utilities. As a result, delivery charges are not published in tariff books, but can be obtained through public information requests.

### Electricity and Natural Gas Charges

Finally, we determine the electricity and natural gas charges for each facility based on the assumption that facilities use primary voltage and opt into time-of-use (TOU) pricing where available. We also assume no unique cogeneration agreements between the energy utility and WWTP, which in practice may lead to a custom tariff. As suggested by Aymerich *et al.*^26^, we ignore reactive charges. A comprehensive list of simplifying assumptions is available in WWTP_Billing_Assumptions.xlsx;^22^ all ex-post analysis is described below.

For Class 1 facilities (fully bundled pre-defined charges), we obtained the charges directly from the selected tariff, which were found in tariff books taken from the energy utility website or public utility commission’s archive. Classes 2–5 require additional assumptions, simplifications, or ex-post analysis. For these classes, we report the sum of the generation, delivery, and fuel cost adjustment charges as a single energy or demand charge. The “Notes” column describes the total electricity energy or demand charge calculation process.

Class 2 facilities have fuel cost adjustments that must be accounted for in ex-post analysis. We obtained the 2021 fuel cost adjustments for each Class 2 facility in addition to the pre-defined tariff (Tables 2–4).

**Table 2:** Data provenance of cogeneration information, electricity tariffs, and natural gas tariffs in WWTP_Billing.xlsx^22^ for wastewater treatment plants with an existing total flow of 50.5 to 75 million gallons per day (MGD) according to CWNS^30^.

CWNS_No	Cogen	Electricity	Natural Gas
12000053001	^21^	^67^	^68^
48003033002	N/A	https://austinenergy.com/rates/commercial-rates	N/A
31001825002	N/A	^69^	N/A
36001010001	N/A	^70^,https://www.psegliny.com/aboutpseglongisland/ratesandtariffs/rateinformation	N/A
36001010017	N/A	^70^,https://www.psegliny.com/aboutpseglongisland/ratesandtariffs/rateinformation	N/A
9000641001	N/A	^71,72^	N/A
6005025001	^21^	^73,74^	^75^
35000021001	^21^	^76^,https://www.pnm.com/fuel-adjustment	^77^, https://www.nmgco.com/en/cost_of_gas
36001010006	N/A	^70^,https://www.psegliny.com/aboutpseglongisland/ratesandtariffs/rateinformation	N/A
48008015001	N/A	^78,79^	N/A
34006012001	^49^	^65^	^80^
6004010004	N/A	^81^	N/A
36002001007	^30^	^82^	^83^
51000161001	N/A	^84,85^	N/A
40000123012	N/A	^86,87^	N/A
53001280001	^45^	^88^	^89^
36002001004	^30^	^82^	^83^
32000011001	N/A	^90^	N/A
36002001006	^30^	^82^	^83^
41000017001	^47^	^91^	^92^
47000245002	N/A	^93,94^	N/A
24000001002	N/A	^95,96^	N/A
42006056001	N/A	^97^	N/A
53000776001	^40^	^98^	^89^
47000940001	N/A	^99^,https://vec.org/service-products/electric/tva-fuel-cost/	N/A
36007136001	^41^	https://www.nationalgridus.com/Upstate-NY-Business/Rates/Service-Rates	https://www.nationalgridus.com/Upstate-NY-Business/Rates/Service-Rates
39002093001	^42^	^100^	^101^
12000001001	^46^	^102^	^103^
10000027001	^50^	^104^	^105^
51000154002	N/A	^84,85^	N/A
6005053001	^48^	^73,74^	^75^

N/A indicates facilities that did not have cogeneration, so no cogen or natural gas data was collected.

**Table 3:** Data provenance of cogeneration information, electricity tariffs, and natural gas tariffs in WWTP_Billing.xlsx^22^ for wastewater treatment plants with an existing total flow of 75.01 to 120 million gallons per day (MGD) according to CWNS^30^.

CWNS_No	Cogen	Electricity	Natural Gas
34001005001	^30^	^65^	^80^
15000003001	^43^	^58–64^	^106^
48004026002	^21^	^107,108^, https://dallascityhall.com/government/citysecretary/openrecords/Pages/test.aspx	^109^
39000084001	^21^	^110^	^111^
36003169012	^39^	^82^	^112^
39001792001	N/A	^113^	N/A
6002032003	^30^	^73,74^	^75^
6002036001	^21^	^73,74^	^75^
26004005011	N/A	^114^	N/A
29001011001	N/A	^57^	N/A
6004009003	N/A	^81^	N/A
34001030001	http://www.jmeuc.com/newsletters.php	^65^	^80^
39008260001	^21^	^115^	^116^
36008024001	N/A	^117,118^	N/A
36002001010	^30^	^82^	^83^
13000012004	^51^	^119,120^	^121^
6008022001	^21^	^81^	^122^
32000200820	N/A	^90^	N/A
47000940002	N/A	^99^,https://vec.org/service-products/electric/tva-fuel-cost/	N/A
47001016001	^30^	^123^,https://vec.org/service-products/electric/tva-fuel-cost/	^124^
12000017028	N/A	^102^	N/A
22009071001	N/A	^125^	N/A
36002001009	^30^	^82^	^83^
42000094003	N/A	^126^	N/A
12000017027	^21^	^102^	^127^
39001792002	N/A	^113^	^116^
48007039001	N/A	https://www.houstontx.gov/pia.html	N/A
36002001005	^30^	^82^	^83^
21000025001	N/A	^128^	N/A
53000776002	^21^	http://www.seattle.gov/city-light/business-solutions/business-billing-and-account-information/business-rates	^89^
29001023001	N/A	^129,130^	N/A
29001023002	N/A	^129,130^	N/A
18000061001	N/A	^131,132^	N/A

N/A indicates facilities that did not have cogeneration, so no cogen or natural gas data was collected.

**Table 4:** Data provenance of cogeneration information, electricity tariffs, and natural gas tariffs in WWTP_Billing.xlsx^22^ for wastewater treatment plants with an existing total flow of more than 120 million gallons per day (MGD) according to CWNS^30^.

CWNS_No	Cogen	Electricity	Natural Gas
36002001002	^30^	^82^	^112^
48004026001	N/A	^107,108^, https://dallascityhall.com/government/citysecretary/openrecords/Pages/test.aspx	N/A
12000017004	^21^	^102^	^127^
36002001003	^30^	^82^	^83^
39001666001	N/A	https://www.cpp.org/info/Pricing/Rate-Schedule	N/A
48004122001	^21^	^107,108^,https://dallascityhall.com/government/citysecretary/openrecords/Pages/test.aspx	^109^
4001318001	^21^	^133^	^134^
6002041001	^21^	^73,74^	^75^
36009071001	N/A	https://www.nationalgridus.com/Upstate-NY-Business/Rates/Service-Rates	N/A
39003369002	N/A	^135,136^	N/A
6008022002	^21^	^81^	^122^
48000004001	N/A	^107,108^,https://dallascityhall.com/government/citysecretary/openrecords/Pages/test.aspx	N/A
8000070001	^30^	^137^	^138^
24000001001	^44^	^95,96^	^139^
42005016001	N/A	^140^	N/A
6005009001	^52^	^141^	^75^
6002121001	^21^	^73,74^	^75^
36002001012	^30^	^82^	^83^
39001666002	N/A	^142^	N/A
34002065001	^21^	^143^	^80^
6009031001	^21^	^144,145^	^146^
42000094001	N/A	^140^	N/A
42000094002	^21^	^140^	^147^
27000001001	N/A	^148^	N/A
55003100001	^21^	^149^	^150^
17000721009	N/A	^151^	N/A
36002001001	^30^	^82^	^112^
36002001011	^30^	^82^	^83^
34001082001	N/A	^65^	N/A
17000721007	N/A	^151^	N/A
25000128001	^21^	^152,153^	^154^
6004010001	^21^	^81^	^122^
6004009001	^21^	^81^	^122^
11000001001	^21^	^155^	^156^
26000596001	N/A	^114^	N/A
17000721001	N/A	^151^	N/A

N/A indicates facilities that did not have cogeneration, so no cogen or natural gas data was collected.

Class 3 facilities have both generation and delivery service from the same provider, but with distinct instead of bundled charges. In that case, the charges were often on different sheets, so the data collection process simply had to be repeated for both generation and delivery charges using the same tariff book.

Class 4 facilities with real-time pricing (RTP) or day-ahead pricing (DAP) in our dataset were limited to NYISO and PJM zones. In cases where WWTPs use RTP or DAP (Table 5), we:

1. Obtained historical 2021 prices from one of two sources, depending on the tariff:

- Real-time prices tied to the locational marginal price (LMP), (e.g., “The real time PJM Load Weighted Average Residual Metered Load Aggregate Locational Marginal Prices for the Public Service Transmission Zone”^65^).
- Day-ahead prices published directly by the utility.

2. Averaged the 2021 historical pricing data according to the existing tariff structure. Since RTP and DAP only existed for generation charges, the pre-defined delivery charges were used as the existing tariff structure. In other words, if the delivery charges were flat, then we calculated a single average generation charge for each month. If there was a TOU component, however, then we calculated a separate average for each TOU period.

**Table 5:** Utilities with real-time pricing for industrial consumers and the corresponding CWNS numbers of the wastewater treatment plants (WWTPs).

Electric Utility	Method	TOU Period	CWNS Numbers	Source
Con Edison	NYISO	Weekdays 8 AM-10 PM	36002001007, 36002001004, 36002001006, 36003169012, 36002001010, 36002001009, 36002001005, 36002001002, 36002001003, 36002001012, 36002001001, 36002001011	https://www.nyiso.com/custom-reports?report=dam_lbmp_zonal
National Grid	DAP	None	36007136001, 36009071001	https://www9.nationalgridus.com/niagaramohawk/home/rates/4_elec_supply.asp
PSEG	PJM	Weekdays 7 AM-9 PM	34006012001, 34001005001, 34001030001, 34001082001	https://www.pjm.com/markets-and-operations/energy/real-time/historical-bid-data
Jersey Central Power & Light	PJM	Weekdays 8 AM-8 PM	34002065001	https://www.pjm.com/markets-and-operations/energy/real-time/historical-bid-data
PECO	PJM	None	42000094001, 42000094002, 42000094003	https://www.pjm.com/markets-and-operations/energy/real-time/historical-bid-data
Duquesne Light Company	PJM	None	42005016001	https://www.pjm.com/markets-and-operations/energy/real-time/historical-bid-data
Penelec	PJM	None	42006056001	https://www.pjm.com/markets-and-operations/energy/real-time/historical-bid-data
Comed	DAP	None	17000721009, 17000721007, 17000721001	https://secure.comed.com/MyAccount/MyBillUsage/Pages/RatesPricing.aspx

PJM = average of PJM locational marginal price, NYISO = average of NYISO locational marginal price, and DAP = average of utility’s day-ahead price.

Class 5 facilities lack published delivery tariffs. In our dataset, Class 5 facilities were limited to Houston and Dallas. Through a public information request, we collected the actual 12-month fixed-rate delivery service contracts for each WWTP and added those delivery charges to the generation charges in the public tariff book to create a comprehensive electricity tariff.

### Dataset Collection Process

The dataset collection process described above can be summarized by the following steps, which were performed independently for each of the 100 WWTPs:

1. Determine electric utility by matching the utility’s service area map with location data from the CWNS^30^.
2. Download the full tariff book for the utility from Step 1.
3. Estimate peak gross electricity demand by plugging in design flow from CWNS into Eq. (1).
4. Determine if the facility has cogeneration based on the decision tree in Fig. 4.

**Fig. 4:**
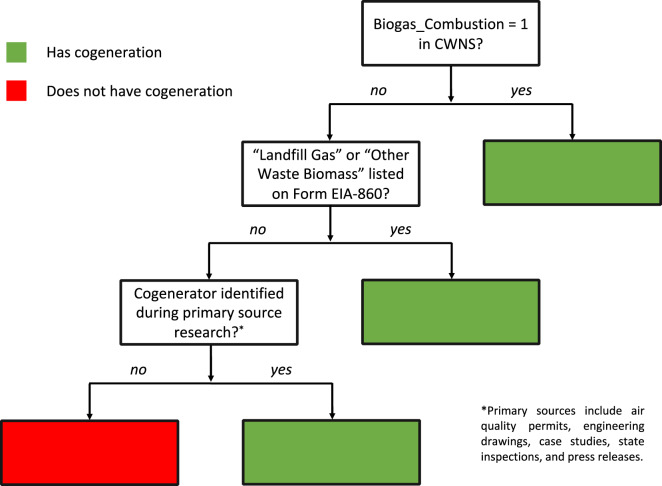
Decision tree used to determine if a facility has on-site cogeneration. First the “Biogas_Combustion” field of the Clean Watershed Needs Survey (CWNS)^30^ was checked, then “Landfill Gas” and “Other Waste Biomass” in Form EIA-860^21^, and finally we performed Google searches to identify primary evidence^22^, such as air quality permits from local^33–35^ and state^36–39^ monitoring authorities, documentation on municipal websites^40–44^, state inspections^45^, technical reports^46^, EPA case studies^47,48^, and press releases^49–52^.
5. Estimate net (i.e., electric grid) demand by plugging D_gross_ from Step 3 and ${\mathbb{1}}$(Cogen) from Step 4 into Eq. (3).
6. Select appropriate electricity tariff from tariff book in Step 2 based on the following criteria: (i) industrial customer; (ii) primary voltage and other simplifying assumptions from WWTP_Billling_Assumptions.xlsx^22^; and (iii) electricity demand from Step 5. An annotated example is provided in Supplementary File 1.
7. Record electricity customer, demand, and energy charges from the tariff book or public information request.

- If necessary, obtain historical real-time or day-ahead pricing to calculate the average electricity generation charge (Table 5).
- If the facility has tiered electricity charges, account for those with a basic charge limit. If there are no tiered charges, all basic charge limits should be zero. A basic charge limit is the quantity of energy delivered (either electricity or natural gas) at which a charge becomes effective.

8. Estimate natural gas demand by plugging D_el_ from Step 5 and ${\mathbb{1}}$(Cogen) from Step 4 into Eq. (4) with f_ng_ = 0.1.
9. If the facility had cogeneration in Step 4, determine natural gas utility by matching the utility’s service area map with location data from the CWNS^30^.
10. If the facility had cogeneration in Step 4, download the full tariff book for the utility from Step 9.
11. Select appropriate natural gas tariff from tariff book in Step 10 based on the following criteria: (i) industrial customer; (ii) simplifying assumptions from WWTP_Billling_Assumptions.xlsx;^22^ and (iii) natural gas demand from Step 8.
12. Record natural gas customer, demand, and energy charges from the tariff book or public information request.
  - If the facility has tiered natural gas charges, account for those with a basic charge limit. If there are no tiered charges, all basic charge limits should be zero.

## Data Records

Data described in this paper are available publicly on figshare^22^. Two Excel spreadsheets and three CSV files are included:

- metadata.csv
- WWTP_Billing.xlsx
- WWTP_Billling_Assumptions.xlsx
- reference_list.csv
- synthetic_energy_data.csv

### Metadata

Metadata is stored in a single CSV file with each facility taking up one row and the following columns named in the header:

- **Index**: Index assigned by authors (1–100)
- **CWNS_No**: CWNS ID number
- **Existing Total Flow (MGD)**: operational flow rate in million of gallons per day according to CWNS
- **Design Flow (MGD)**: design flow rate in million gallons per day according to CWNS
- **Existing Total Flow (m3/day)**: operational flow rate in cubic meters per day according to CWNS (using a conversion factor of 3785.41178 cubic meters = 1 million gallons)
- **Design Flow (m3/day)**: design flow rate in cubic meters per day according to CWNS (using a conversion factor of 3785.41178 cubic meters = 1 million gallons)
- **City**: city where the facility is located, e.g., “Syracuse”
- **County**: county where the facility is located, e.g., “Onondaga”
- **State**: two-letter code for the state where the facility is located, e.g., “NY”
- **Latitude**: “latitude decimal degree for a point. Positive values are used for both Northern and Southern hemisphere and have to be used in conjunction with the Latitude direction. The measure of the degree portion of a latitude measurement (0 to 90 degrees), indicating angular distance North or South of the equator. One degree of latitude equals 111.1 Kilometers or approximately 60 Nautical Miles. Includes the direction of the latitude measurement, being either: N – North, or S – South”^30^.
- **Longitude**: “longitude decimal degree for a point. Positive values are used for both Eastern and Western hemisphere and have to be used in conjunction with the Longitude direction. The measure of the degree portion of longitude (000 to 180 degrees), indicating angular distance West or East of the prime meridian drawn from pole to pole around the Earth and passing through Greenwich, England. Includes the direction of the longitude measurement being either: E – East, or W – West”^30^.
- **Horizontal Collection Method**: “text that describes the method used to determine the latitude and longitude coordinates for a point on the earth”^30^
- **Horizontal Coordinate Datum**: name of the reference datum used in determining latitude and longitude coordinates from CWNS^30^. The options are North American Datum of 1983 or World Geodetic System of 1984
- **Location Description**: name of the place where the coordinates were measured (taken directly from CWNS^30^). The options are: Lagoon or Settling Pond; Facility/Station Location; Intake/Release Point; Treatment/Storage Point; or Center/Centroid
- **Location Source**: Indicates how the point was entered. “Manual” indicates that the state user entered the coordinate information. “NPDES Permit” indicates that the coordinates were sourced from the information provided on the NPDES permit^30^
- **Scale**: Scale: text that describes the geopositioning or scale of the map used by CWNS^30^, e.g., 1:1000
- **Has Cogen**: whether the facility has onsite cogeneration (regardless of whether they are operating it). Either “Yes” or “No”
- **Est. Existing Electricity Demand (MW)**: estimated average gross electricity demand in MW. Calculated using Existing Total Flow (MGD) in Eq. (1)
- **Est. Existing Electric Grid Demand (MW)**: estimated average electric grid demand in MW. Calculated using Existing Total Flow (MGD) in Eq. (3)
- **Est. Existing Natural Gas Demand (therms/hr)**: estimated average natural gas demand in therms per hour. Calculated using Existing Total Flow (MGD) in Eq. (4)
- **Est. Existing Natural Gas Demand (m3/hr)**: estimated average natural gas demand in cubic meters per hour (using a conversion factor of 2.83168 cubic meters = 1 therm). Calculated using Existing Total Flow (MGD) in Eq. (4)
- **Est. Design Electricity Demand (MW)**: estimated maximum gross electricity demand in MW. Calculated using Design Flow (MGD) in Eq. (1)
- **Est. Design Electric Grid Demand (MW)**: estimated maximum electric grid demand in MW. Calculated using Design Flow (MGD) in Eq. (3)
- **Est. Design Natural Gas Demand (therms/hr)**: estimated maximum natural gas demand in therms/hr. Calculated using Design Flow (MGD) in Eq. (4)
- **Est. Design Natural Gas Demand (m3/hr)**: estimated maximum natural gas demand in cubic meters per hour (using a conversion factor of 2.83168 cubic meters = 1 therm). Calculated using Design Flow (MGD) in Eq. (4)
- **Electric Utility**: electric utility, e.g., “National Grid”
- **Gas Utility**: natural gas utility, e.g., “National Grid”
- **Electricity Energy Charge Temporality**: categorization of the temporality of electricity energy charges. “Flat” indicates charges are constant throughout the year; “Seasonal-TOU” indicates charges vary monthly (Seasonal) and daily and/or hourly (TOU); “Nonseasonal-TOU” indicates charges are consistent from month to month, but vary daily and/or hourly; and “Seasonal-NonTOU” indicates charges vary monthly, but are constant from day to day and hour to hour
- **Electricity Demand Charge Temporality**: categorization of the temporality of electricity demand charges. “Flat” indicates charges are constant throughout the year; “Seasonal-TOU” indicates charges vary monthly (Seasonal) and daily and/or hourly (TOU); “Nonseasonal-TOU” indicates charges are consistent from month to month, but vary daily and/or hourly; and “Seasonal-NonTOU” indicates charges vary monthly, but are constant from day to day and hour to hour

Table 6 shows metadata provenance. Data on a facility’s flow rate, city, state, and county directly from the CWNS^30^ was supplemented with the previously described procedure for determining whether a facility has cogeneration. Descriptions of utility service areas were used to determine the correct utility for each municipality.

**Table 6:** Data provenance of metadata.csv^22^.

Attribute	Source
Index	Assigned by authors
Index in Initial List	^30^
CWNS_No	^30^
Existing Total Flow (MGD)	^30^
Design Flow (MGD)	^30^
Existing Total Flow (m3/day)	Converted “Existing Total Flow (MGD)” to SI units
Design Flow (m3/day)	Converted “Design Flow (MGD)” to SI units
City	^30^
County	^30^
State	^30^
Latitude	^30^
Longitude	^30^
Horizontal Coordinate Datum	^30^
Horizontal Collection Method	^30^
Location Description	^30^
Location Source	^30^
Scale	^30^
Has Cogen	Varies by facility, see reference_list.csv^22^
Est. Existing Energy Demand (MW)	Calculated using “Existing Total Flow (MGD)” in Eq. (1)
Est. Existing Electric Grid Demand (MW)	Calculated using “Existing Total Flow (MGD)” in Eq. (2)
Est. Existing Natural Gas Demand (therms/hr)	Calculated using “Existing Total Flow (MGD)” in Eq. (4)
Est. Existing Natural Gas Demand (m3/hr)	Converted “Est. Existing Natural Gas Demand (therms/hr)” to SI units
Est. Design Energy Demand (MW)	Calculated using “Design Flow (MGD)” in Eq. (1)
Est. Design Natural Gas Demand (therms/hr)	Calculated using “Design Flow (MGD)” in Eq. (4)
Est. Design Natural Gas Demand (m3/hr)	Converted “Est. Design Natural Gas Demand (therms/hr)” to SI units
Electricity Utility	Varies by facility, see reference_list.csv^22^
Gas Utility	Varies by facility, see reference_list.csv^22^
Electricity Energy Charge Temporality	Assigned by authors. See Categorization of Electricity and Natural Gas Tariffs
Electricity Demand Charge Temporality	Assigned by authors. See Categorization of Electricity and Natural Gas Tariffs

### Billing Data

Each worksheet of WWTP_Billing.xlsx^22^ gives the name of the CWNS number corresponding to a facility. Each row corresponds to a different charge, so a municipality with a flat electricity tariff would have only one charge and therefore one row, whereas a municipality with a complex tariff would have many rows corresponding to many charges. Natural gas tariffs are included only for facilities that have cogeneration; without cogeneration the natural gas use is minimally changed by participating in energy flexibility programs^37^. An annotated data sample is provided in Supplementary File 2. The electricity and natural gas tariff data has the following columns:

- **utility**: type of utility, i.e., “electric” or “gas”
- **type**: type of charge. Options are “customer”, “demand”, and “energy”
- **period**: name for the charge period. Only relevant for demand charges, since there can be multiple concurrent demand charges. E.g., a charge named “maximum” that is in effect 24 hours a day vs. a charge named “on-peak” that is only in effect during afternoon hours.
- **basic_charge_limit (imperial)**: consumption limit above which the charge takes effect in imperial units (i.e., kWh of electricity and therms of natural gas). Default is 0. A limit is in effect until another limit supersedes it. E.g., if there are two charges, Charge 1 with basic_charge_limit = 0 and Charge 2 with basic_charge_limit = 1000, Charge 1 will be in effect until 1000 units are delivered, and Charge 2 will be in effect thereafter.
- **basic_charge_limit (metric)**: consumption limit above which the charge takes effect in metric units (i.e., kWh of electricity and m^3^ of natural gas). Default is 0. A limit is in effect until another limit supersedes it. E.g., if there are two charges, Charge 1 with basic_charge_limit = zero and Charge 2 with basic_charge_limit = 1000, Charge 1 will be in effect until 1000 units are delivered, and Charge 2 will be in effect thereafter.
- **month_start**: first month during which this charge occurs (1–12)
- **month_end**: last month during which this charge occurs (1–12)
- **hour_start**: hour at which this charge starts (0–24)
- **hour_end**: hour at which this charge ends (0–24)
- **weekday_start**: first weekday on which this charge occurs (0 = Monday to 6 = Sunday)
- **weekday_end**: last weekday on which this charge occurs (0 = Monday to 6 = Sunday)
- **charge (imperial)**: cost represented as a float in imperial units. I.e., “$/month”, “$/kWh”, “$/kW”, “$/therm”, and “$/therm/hr” for customer charges, electricity energy charges, electric demand charges, natural gas energy charges, and natural gas demand charges, respectively
- **charge (metric)**: cost represented as a float in metric units. I.e., “$/month”, “$/kWh”, “$/kW”, “$/m3”, and “$/m3/hr” for customer charges, electricity energy charges, electricity demand charges, natural gas energy charges, and natural gas demand charges, respectively. A conversion factor of 2.83168 cubic meters to 1 therm was used.
- **units**: units of the charge, e.g. “$/kWh”. If units are different between imperial and metric then imperial is listed followed by metric. E.g., “$/therm or $/m3”.
- **Notes**: any comments the authors felt would help explain unintuitive decisions in data collection or formatting

### Billing Assumptions

Besides the assumptions made for every facility laid out in the Methods section, some utilities had nuanced tariffs that required further simplifying assumptions. These assumptions are catalogued in WWTP_Billing_Assumptions.xlsx, which has two worksheets: ‘Electric’ and ‘Gas’. Both have a new assumption on each row and identical columns:

- **Assumptions**: explanation of the assumption the authors made in determining the correct tariff for these facilities
- **CWNS_No_1, CWNS_No_2, …, CWNS_No_14**: list of facilities for which this assumption applies. “ALL” indicates the assumption was applied to all facilities.

### Reference List

For posterity, we saved captures of all the original tariffs and indexed them in reference_list.csv, which includes the following information:

- **Document Title**: name of the document being referenced
- **Utility**: utility that published this tariff
- **Day Filed**: day this tariff was filed (if known)
- **Month Filed**: month this tariff was filed (if known)
- **Year Filed**: year this tariff was filed (if known)
- **Day Effective**: day this tariff became effective (if known)
- **Month Effective**: month this tariff became effective (if known)
- **Year Effective**: year this tariff became effective (if known)
- **Day Accessed**: day this tariff was downloaded (if known)
- **Month Accessed**: month this tariff was downloaded (if known)
- **Year Accessed**: year this tariff was downloaded (if known)
- **Relevant CWNS Numbers**: list of facilities to which this tariff applies

If the referenced tariff included multiple sheets with different filed or effective dates, then the most recent date of the entire book was used. Date accessed is only included for non-static HTML pages, which were archived with the Wayback Machine (https://archive.org/web/). The original versions of all documents are also available on figshare^22^. A copy of the information available in reference_list.csv is separated by existing total flow of 50.5–75 MGD (Table 2), 75.01-120 MGD (Table 3), and >120 MGD (Table 4).

### Synthetic Energy Data

For our sample case study, we include synthetic energy data for a WWTP. One week of sample energy data at a 15-minute timescale is copied for a year to be used in the sample analysis (see Usage Notes):

- **DateTime**: date and time of synthetic energy data sample
- **grid_to_plant_kW**: electricity delivered from the grid in kW
- **natural_gas_therm_per_hr**: natural gas delivered to the cogenerator in therms/hr

## Technical Validation

### Global Checks

Seven global checks were performed to ensure completeness, i.e., all timesteps are covered, and reasonableness, i.e., all values are within reasonable bounds:

1. average flow is increasing with each facility and is within bounds of smallest/largest flows;
2. natural gas tariffs are included if and only if a facility has cogeneration;
3. prices are positive and below a reasonable threshold (e.g., $0.50 / kWh);
4. all hours/days of the year are included;
5. units are correct (i.e. demand is in kW, therms/hr, or m^3^/hr and energy in kWh, therms, or m^3^);
6. correct conversion factors were used (i.e. 2.831681 m^3^ per therm of natural gas and 3785.41178 m^3^ per million gallons); and
7. Equations (1–4) hold.

The maximum thresholds for prices were set manually at the start and then each exception to the threshold was double-checked for correctness in the original tariff document.

### Validation with Existing Data

We compared the metadata collected here to wastewater treatment plant data collected by Chini & Stillwell^66^ determine the accuracy of our data collection procedure. Chini & Stillwell’s data was collected at a city-level through public information requests in 2012. Data was reported on a monthly basis for treatment volume (MG), electricity consumption (kWh), natural gas consumption (therms), and biogas consumption (therms). Data for natural gas and biogas consumption was not available for all cities.

Since Chini & Stillwell’s dataset is compiled at a city-level, the first step was selecting WWTPs that were the only facility in their municipality. I.e., we ensured there was a one-to-one correspondence between the WWTP and the city by verifying that the city’s treatment volume^66^ was within 10% of the facility’s existing total flow or design flow reported in the CWNS^30^. This gave us nine cities to use for validation (Table 7).

**Table 7:** Comparison between our dataset and Chini & Stillwell (2018)^66^ for validation purposes.

CWNS No.	City	Avg. Volume (MGD)^66^	Existing Total Flow (MGD)^30^	Design Flow (MGD)^30^	Avg. Electricity (MW)^66^	Est. Existing Electric Grid Demand (MW)	Est. Design Electricity Demand (MW)
12000053001	Tampa	59.78	50.5	96	6.33	2.63	5
48008015001	San Antonio	125.29	58	125	8.97	6.04	13.02
36007136001	Syracuse	75.45	71.153	80	7.26	3.71	4.17
22009071001	New Orleans	102.00	92.10	122	3.52	9.59	10.1
53000776002	Seattle	102.48	110	215	6.01	5.73	11.2
4001318001	Phoenix	170.54	140	230	11.80	7.29	11.98
42005016001	Pittsburgh	183.64	164	250	9.68	17.08	26.04
6009031001	San Diego	171.90	184	240	11.97	9.58	12.5
25000128001	Boston	292.59	310	310	14.72	16.15	16.15

These 9 cities were selected as they were (i) found in both datasets and (ii) had similar flow rates indicating that a single facility treated all of the city’s wastewater.

The discrepancies in electricity demand are acceptable for the purpose of this paper, which was to assign the appropriate electricity or natural gas tariff to each facility. For example, we overestimate the electricity demand in New Orleans and Pittsburgh while underestimating demand in Syracuse and Atlanta. Across the 9 matching WWTPs, Est. Design Electric Grid Demand (MW) was 13.2% higher on average than the electricity demand from Chini & Stillwell^66^, indicating that our estimation methodology is reasonable.

With that said, we propose two explanations for the discrepancies between the two datasets. First, Chini & Stillwell collected monthly data, so the electricity demand computed is an average monthly demand. However, our dataset attempts to estimate *peak* electricity demand for use in determining electricity and natural gas tariffs. Second, we use a constant energy intensity factor to approximate the energy demand of wastewater treatment. In reality, economies of scale will lead to an energy intensity factor that declines with treatment volume.

As stated in the Methods section, varying f_ng_, the fraction of natural gas blended with biogas during cogeneration between 4.4% and 40% did not change the natural gas tariff selected for any facility. Similarly, using existing total flow instead of design flow from the CWNS database (which is often a large discrepancy) would lead to a change in only 4 of 100 WWTPs’ electricity tariffs. In conclusion, these small over/underestimates in electricity or natural gas demand do not impact the correct tariff selection.

Having validated our electricity and natural gas demand estimation methods, we directly contacted a handful of facilities to verify that the tariff we assigned to them was correct. We were able to reach facilities through our existing research network in California, manually verifying that our tariffs for Pacific Gas & Electric, Southern California Edison, and Southern California Gas were appropriate for WWTPs in those service areas.

## Usage Notes

**Fig. 5:**
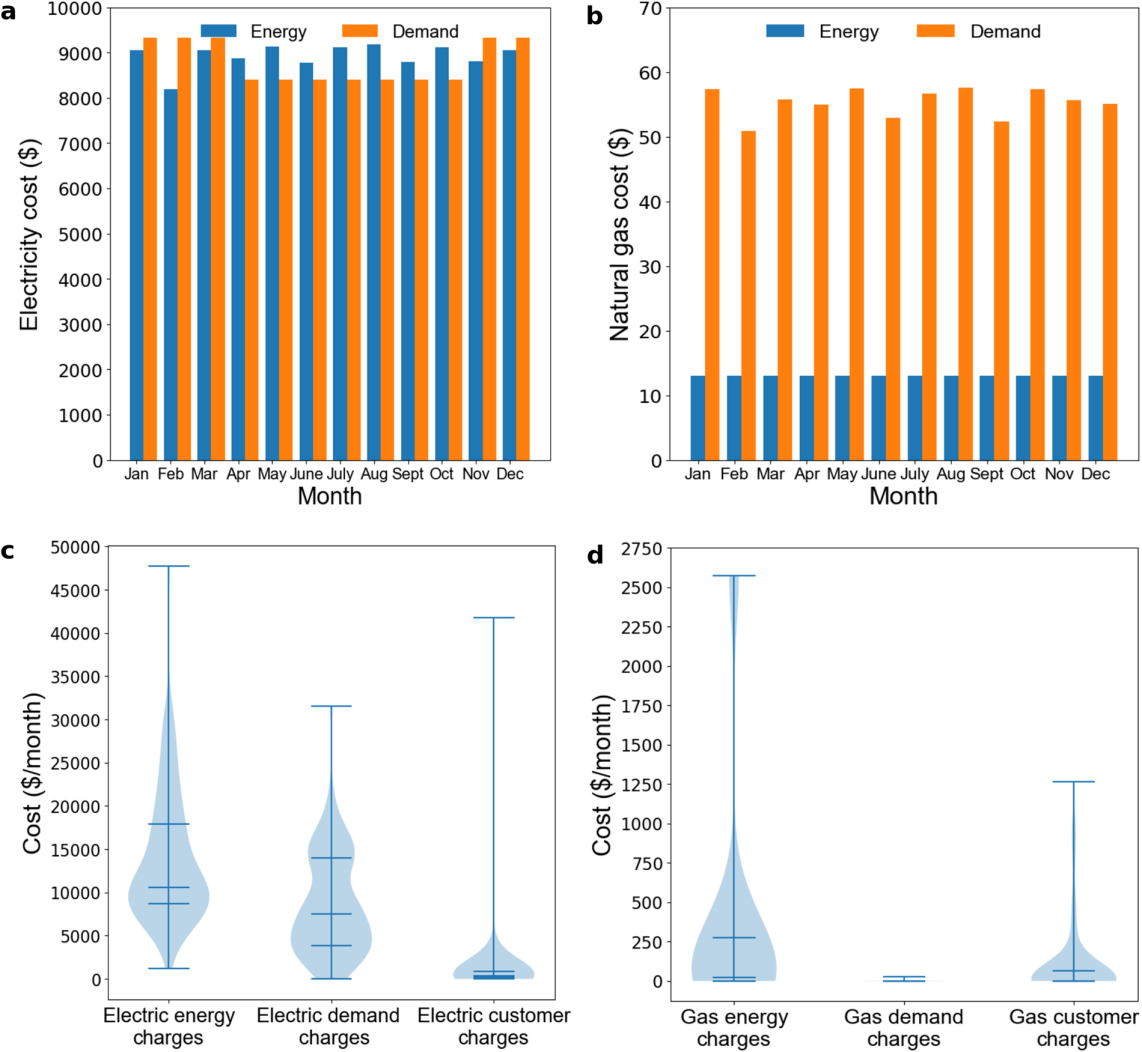
Sample results from simulating the estimated annual cost of electric power and natural gas charges from the 100 WWTPs in our dataset**. (a)** Monthly electricity energy and demand charges were simulated using synthetic consumption data modeled on a case study facility in California and the energy and natural gas tariffs associated with CWNS Facility No. 12000017027. **(b)** Natural gas energy and demand charges were simulated using synthetic consumption data and the tariff associated with CWNS Facility No. 12000017027. Customer charges were not plotted since they do not vary month-to-month. (**c)** Violin plot showing the distribution of electricity costs for each charge type from an annual simulation of all 100 facilities using synthetic data. Horizontal lines indicate the minimum, first quartile, second quartile, third quartile, and maximum from bottom to top. **(d)** Violin plot showing the distribution of natural gas costs for each charge type from an annual simulation of all 100 facilities using synthetic data. Horizontal lines indicate the minimum, first quartile, second quartile, third quartile, and maximum from bottom to top.

One week of case study facility data at 15-minute timescales was repeated for a year to create a synthetic energy consumption dataset. Using that dataset and sample code, which are both available on GitHub (https://github.com/we3lab/wwtp-energy-tariffs), we conducted an energy cost simulation. CWNS Facility No. 12000017027 was selected for this example because it has the full range of charge types (customer, electricity demand, electricity energy, natural gas demand, and natural gas energy). The results of the simulation are shown in Fig. 5a,b. Customer charges of $283.03/mo for electricity and $300.00/mo for natural gas were not plotted since they are identical from month-to-month. Analogous simulations were run on all 100 facilities to verify the dataset’s completeness (Fig. 5c,d).

### Supplementary information

Supplementary File 1

Supplementary File 2

## Data Availability

The data is provided as Excel and CSV spreadsheets that can be used without code for manipulation. Sample Python scripts are available on GitHub to ease analysis and demonstrate technical validation procedures (https://github.com/we3lab/wwtp-energy-tariffs).
