# Supplementary material for: Electricity and natural gas tariffs at United States wastewater treatment plants
Source: Sci Data. 2024 Jan 23;11:113. doi: 10.1038/s41597-023-02886-6 (PMC10805726; doi:10.1038/s41597-023-02886-6)
Supplement: Supplementary file 1 [file 41597_2023_2886_MOESM1_ESM.pdf]

Annotated electricity tariff selection for CWNS Facility No. 47001016001 in Nashville, TN.

ELECTRIC POWER BOARD OF THE METROPOLITAN GOVERNMENT OF NASHVILLE AND DAVIDSON COUNTY

LARGE GENERAL POWER RATE SCHEDULES

(November 2021)

Availability

These rates shall apply to the firm electric power requirements where a customer's currently effective onpeak or offpeak contract demand, whichever is higher, is greater than 5,000 kW; provided that the other conditions of this section are met.

Unless otherwise provided for in a written agreement between TVA and the distributor providing service under these rate schedules, for customers served under these rate schedules, the customer's "meter-reading time" shall be 0000 hours CST or CDT, whichever is currently effective, on the first day of the calendar month following the month for which a bill under these rate schedules is being calculated. Further, in accordance with TVA-furnished or TVA-approved guidelines or specifications, TVA shall have unrestricted remote access to the metering data at all times, as well as unrestricted physical access to the metering facilities for the purpose of confirming remotely-accessed data during such periods as are specified by TVA.

For a customer requesting that its onpeak contract demand be different from its offpeak contract demand, these rate schedules shall be available only for (1) a new contract, (2) a replacement or renewal contract following expiration of the existing contract, or (3) a replacement or renewal contract or an amended existing contract in which the customer is increasing its demand requirements above the existing contract demand level, but under this item (3) neither the new onpeak nor the new offpeak contract demand shall be lower than the customer's existing contract demand

The General Power Rate--Schedule GSB rate schedule shall apply to customers with an onpeak or offpeak contract demand, whichever is higher, greater than 5,000 kW, but not more than 15,000 kW; The General Power Rate--Schedule GSC rate schedule shall apply to customers with an onpeak or offpeak contract demand, whichever is higher, greater than 15,000 kW, but not more than 25,000 kW; and the General Power Rate--Schedule GSD rate schedule shall apply to customers with an onpeak or offpeak contract demand, whichever is higher, greater than 25,000 kW.

(a) Since metadata.csv shows CWNS Facility No. 47001016001 had Est. Design Electric Grid Demand of 6.5 MW, Schedule GSB would be the appropriate tariff

Base Charges Attachment

	Schedule GSB	Schedule GSC	Schedule GSD	
Service Charge	$2,000	$2,000	$2,000	per delivery point per month
Administrative Charge	$350	$350	$350	per delivery point per month

Demand Charges:

		Schedule GSB ($/kW)	Schedule GSC ($/kW)	Schedule GSD ($/kW)	
Summer Period	Onpeak Demand	10.87	10.87	10.87	per month of onpeak billing demand
	Maximum Demand	5.38	5.38	5.37	per month of maximum billing demand
	Excess Demand	10.87	10.87	10.87	per month of the amount, if any, by which (1) the customer's onpeak billing demand exceeds its onpeak contract demand or (2) the customer's offpeak billing demand exceeds its offpeak contract demand, whichever is higher.
Winter Period	Onpeak Demand	9.90	9.90	9.90	per month of onpeak billing demand
	Maximum Demand	5.38	5.38	5.37	per month of maximum billing demand
	Excess Demand	9.90	9.90	9.90	per month of the amount, if any, by which (1) the customer's onpeak billing demand exceeds its onpeak contract demand or (2) the customer's offpeak billing demand exceeds its offpeak contract demand, whichever is higher.
Transition Period	Onpeak Demand	9.90	9.90	9.90	per month of onpeak billing demand
	Maximum Demand	5.38	5.38	5.37	per month of maximum billing demand
	Excess Demand	9.90	9.90	9.90	per month of the amount, if any, by which (1) the customer's onpeak billing demand exceeds its onpeak contract demand or (2) the customer's offpeak billing demand exceeds its offpeak contract demand, whichever is higher.

Determination of Seasonal Periods

Summer Period shall mean the June, July, August, and September billing months. Winter Period shall mean the December, January, February, and March billing months. Transition Period shall mean the April, May, October, and November billing months.

Determination of Onpeak and Offpeak Hours

Except for Saturdays, Sundays, November 1, and the weekdays that are observed as Federal holidays for New Year's Day, Memorial Day, Independence Day, Labor Day, Thanksgiving Day, and Christmas Day, Onpeak hours for each day shall for purposes of these rate schedules be from 1 p.m. to 7 p.m. during the months of April, May, June, July, August, September and October and from 4 a.m. to 10 a.m. during the months of January, February, March, November, and December. For all other hours of each day and all hours of such excepted days shall be offpeak hours. Such times shall be Central Standard Time or Central Daylight Time, whichever is then in effect. Said onpeak and offpeak hours are subject to change by TVA. In the event TVA determines that such changed onpeak and offpeak hours are appropriate, it shall so notify Distributor at least 12 months prior to the effective date of such changed hours.

(b) Seasonal and time-of-use (TOU) periods are defined later in the tariff book.
