# Supplementary material for: Electricity and natural gas tariffs at United States wastewater treatment plants
Source: Sci Data. 2024 Jan 23;11:113. doi: 10.1038/s41597-023-02886-6 (PMC10805726; doi:10.1038/s41597-023-02886-6)
Supplement: Supplementary file 2 [file 41597_2023_2886_MOESM2_ESM.pdf]

Annotated data sample from WWTP_Billing.xlsx for CWNS Facility No. 47001016001.

utility	type	period	basic_charge_limit (imperial)	basic_charge_limit (metric)	month_start	month_end	hour_start	hour_end	weekday_start	weekday_end	charge (imperial)	charge (metric)	units	Notes
electric	customer										2350	2350	$/month	
electric	energy		[illegible]	0	1	3	0	4	0	4	0.06514	0.06514	$/kWh	
electric	energy		[illegible]	0	1	3	4	10	0	4	0.07648	0.07648	$/kWh	
electric	energy		[illegible]	0	1	3	10	24	0	4	0.06514	0.06514	$/kWh	
electric	energy		[illegible]	0	1	3	0	24	5	6	0.06514	0.06514	$/kWh	
electric	energy		[illegible]	0	4	5	0	24	0	6	0.0626	0.0626	$/kWh	
electric	energy		[illegible]	0	6	9	0	13	0	4	0.06292	0.06292	$/kWh	
electric	energy		[illegible]	0	6	9	13	19	0	4	0.08783	0.08783	$/kWh	
electric	energy		[illegible]	0	6	9	20	24	0	4	0.06292	0.06292	$/kWh	
electric	energy		[illegible]	0	6	9	0	24	5	6	0.06292	0.06292	$/kWh	
electric	energy		[illegible]	0	10	11	0	24	0	6	0.0626	0.0626	$/kWh	
electric	energy		[illegible]	0	12	12	0	4	0	4	0.06514	0.06514	$/kWh	
electric	energy		[illegible]	0	12	12	4	10	0	4	0.07648	0.07648	$/kWh	
electric	energy		[illegible]	0	12	12	10	24	0	4	0.06514	0.06514	$/kWh	
electric	energy		0	0	12	12	0	24	5	6	0.06514	0.06514	$/kWh	
electric	demand	winter-peak1	0	0	1	3	4	10	0	4	9.9	9.9	$/kW	
electric	demand	transition-peak1	0	0	4	5	13	19	0	4	9.9	9.9	$/kW	
electric	demand	summer-peak	0	0	6	9	13	19	0	4	10.87	10.87	$/kW	
electric	demand	transition-peak1	0	0	10	10	13	19	0	4	9.9	9.9	$/kW	
electric	demand	winter-peak2	0	0	11	12	4	10	0	4	9.9	9.9	$/kW	
electric	demand	maximum	0	0	1	12	0	24	0	6	5.38	5.38	$/kW	
gas	customer										800	800	$/month	
gas	demand	maximum	0	0							0.8	0.283	$/therm/hr or $/m3/hr	
gas	energy		0	0	1	12	0	24	0	6	0.187	0.066	$/therm or $/m3	
gas	energy		15000	42475.2	1	12	0	24	0	6	0.168	0.059	$/therm or $/m3	
gas	energy		40000	113267.2	1	12	0	24	0	6	0.14	0.049	$/therm or $/m3	
gas	energy		90000	254851.2	1	12	0	24	0	6	0.08	0.028	$/therm or $/m3	

(a) Customer charges are a flat monthly fee. Demand charges are assessed per kW, therm/hr, or m^3/hr of peak monthly load during the specified time period. Energy charges are assessed by cumulative consumption (in kWh, therms, or m^3) over the month during the specified time period

(b) Multiple demand charges are distinguished by “period”. Non-contiguous hours for the same billing period can be specified using the same “period” identifier

(c) weekend_start/end and month_start/end are an inclusive range from 0 = Monday to 6 = Sunday and 1 = January to 12 = December, respectively. hour_start is included in the range, but hour_end is excluded. I.e., the first charge applies from 4 AM to 10 AM, and the second from 10 AM to midnight Monday to Friday in January, February, and March

(d) After 15000 therms (or 4247.5 m^3) of natural gas are delivered the price goes from $0.187/therm ($0.066/m^3) to $0.168/therm ($0.059/m^3)
